# Quantitative and functional changes in platelets and fibrinogen following cardiopulmonary by-pass in children

**DOI:** 10.3389/fped.2024.1453182

**Published:** 2024-09-17

**Authors:** Margherita Plebani, David Longchamp, Pauline Lauwers, Stefano Di Bernardo, Maria-Helena Perez

**Affiliations:** ^1^ Pediatric Infectious Diseases and Vaccinology Unit, Department Women-Mother-Child, Lausanne University Hospital and University of Lausanne, Laussanne, Switzerland; ^2^ Pediatric Intensive Care Unit, Department Woman-Mother-Child, Lausanne University Hospital and University of Lausanne, Laussanne, Switzerland; ^3^ Pediatric Cardiology, Department Woman-Mother-Child, Lausanne University Hospital and University of Lausanne, Laussanne, Switzerland

**Keywords:** platelet, fibrinogen, ROTEM, cardiopulmonary bypass, coagulopathy, children, congenital heart disease

## Abstract

**Introduction:**

Cardiopulmonary bypass (CPB) causes coagulopathy, increasing the risk of postoperative bleeding and mortality. The underlying causes of post-CPB coagulopathy and the factors associated with its occurrence are not yet fully understood. This study assesses platelet and fibrinogen concentration and function following CPB in children with congenital heart diseases (CHD).

**Methods:**

We analyzed prospective data from 104 patients aged 0–16 years who underwent CPB surgery for CHD. Blood samples were collected before surgery and within 30 min of CPB completion. In addition to usual coagulation tests, functional analyses were performed using point of care systems with thromboelastometry and impedance aggregometry.

**Results:**

Platelet count, fibrinogen concentration, and platelet and fibrinogen activities significantly decreased after CPB. The duration of CPB was directly associated with a reduction in platelet count and fibrinogen level (*r* = −0.38, *p* < 0.001; *r* = −0.21, *p* = 0.03, respectively), but not with their measured activity. Postoperative percentages of baseline values for platelet count (58.36% [43.34–74.44] vs. 37.44% [29.81–54.17], *p* < 0.001) and fibrinogen concentration (73.68% [66.67–82.35] vs. 65.22% [57.89–70.83], *p* < 0.001) were significantly higher in patients who did not experience hypothermia during surgery. Age was inversely associated with the decrease in platelet count (*r* = 0.63, *p* < 0.001), TRAPTEM AUC (*r* = 0.43, *p* < 0.001), fibrinogen concentration (*r* = 0.44, *p* < 0.001) and FIBTEM MCF (*r* = 0.57, *p* < 0.001).

**Conclusion:**

Post-CPB coagulopathy is multifactorial and not solely attributed to hemodilution. It also involves functional changes in coagulation cascade components, which can be demonstrated by thromboelastometry and impedance aggregometry. Young children, patients requiring prolonged CPB surgery, or those experiencing hypothermia are particularly affected.

## 1 Introduction

Cardiac surgery performed under cardiopulmonary bypass (CPB) induces a coagulopathy, potentially resulting in bleeding and increased postoperative morbidity and mortality. Postoperative bleeding is a significant challenge in cardiac surgery and is more frequent and important in children compared to adults (1). Bleeding within the initial postoperative hours is independently associated with higher mortality rates, prolonged mechanical ventilation, extended intensive care and hospital stays, as well as an increased risk of readmission within 30 days (2, 3).

The causes of postoperative bleeding in pediatric cardiac surgery are multifactorial. They include surgical factors related to the technical complexity of these invasive procedures and hemostasis issues. Hemostasis is a complex process involving platelet activity, coagulation factors, and a fibrinolytic system. Factor's coagulation concentrations are different in infants, reaching those of adults between a few weeks and six months of life (1). While fibrinogen and platelet values do not significantly differ from those in adults, reduced platelet activity has been observed during the first weeks of life (1). The progressive evolution observed in the functional and quantitative aspects of the coagulation system from the neonatal period through adolescence is referred to as “developmental hemostasis” (4). Heart surgery with CPB leads to hemodilution with the various solutes required to fill the CPB machine's circuits (priming). Priming can be performed with either blood products (whole blood or erythrocyte concentrate and fresh frozen plasma) or crystalloids. The hemodilution effect is more pronounced when using crystalloids, as blood products contain coagulation factors. The blood then circulates extravascularly, in contact with external surfaces such as tubes, connections, and membrane oxygenators. This process can affect the three components of hemostasis through several mechanisms. The priming of the circuit causes a reduction in blood cell count and coagulation factors, especially in young children, due to the larger size of the circuit compared to their blood volume (5, 6).

However, post-CPB hemorrhagic diathesis cannot be exclusively explained by this phenomenon, which is partially reversible with blood product supplementation (5). Blood contact with the bypass circuit and the resulting systemic inflammatory response activates the hemostatic system and fibrinolysis, resulting in factor consumption and hyporeactivity to stimulation. Conditions that have been associated with a greater bleeding diathesis are low body weight, age under 12 months, hypothermia during CPB, cyanotic congenital heart disease (CHD), and prolonged CPB duration (1, 7–10).

In recent years, thromboelastometry and impedance aggregometry have been used to study functional aspects of coagulation, including in pediatric patients undergoing CPB (7, 10–12). These tools enable the assessment of different hemostatic response patterns, using different activators and studying the quality of the hemostatic response. Moreover, when available as point-of-care tests, they provide faster results than conventional coagulation tests, making them an attractive tool in clinical practice, although their use in pediatrics still needs to be standardized (13, 14).

In summary, the understanding of post-CPB coagulopathy in pediatric patients remains limited. Therefore, we aimed to investigate the variables influencing platelet count and fibrinogen concentration and activity in this context.

## 2 Method

### 2.1 Study design

This study is a secondary analysis of data collected during the POCHEMO prospective cohort study (15, 16). It was a prospective, observational study of hemostasis in children with CHD. It was a monocentric study conducted at the University Hospital of Lausanne in Switzerland. The local Ethics Committee approved the POCHEMO study protocol (protocol 168/14).

### 2.2 POCHEMO cohort

The POCHEMO database contains data from 200 patients. Inclusion was offered to all patients between 0 and 16 years old with CHD who underwent cardiac catheterization or heart surgery between March 2015 and July 2017 at Lausanne University Hospital. Written consent was obtained from all participants’ legal representatives. Patients with known coagulopathies and those who had received heparin, oral anticoagulants, or antiplatelet agents within 6 h, 48 h, or ten days, respectively, prior to the procedure were excluded. All patients had a complete blood count (CBC), routine coagulation tests, thromboelastometry, and impedance aggregometry analysis before and after surgery. Bleeding and administration of blood products during and up to 6 h after surgery were recorded.

### 2.3 Secondary analysis

For this study, we focused only on patients who underwent cardiac surgery. We excluded patients who received platelet or fibrinogen concentrate transfusion during CPB and patients with missing pre-and/or postoperative platelet count and/or fibrinogen concentration values.

### 2.4 Surgical procedure and CPB

Procedures were performed under general anesthesia, heparinized CPB, and normothermia or hypothermia (defined as core temperature below 35°C) (17). The priming solution for the CPB circuit was either erythrocyte concentrate and fresh frozen plasma or crystalloid, depending on the estimated circulating volume of the child. At the beginning of CPB, patients were anticoagulated with 300 IU/kg of heparin, and anticoagulation was reversed with protamine at the end of CPB. Fluid balance was recorded when weaning from CPB. The anaesthetist did not have access to the analyses conducted for the study.

### 2.5 Blood sampling

Blood samples were taken during anesthesia, before heparin administration, and within 30 min after CPB termination. Arterial or central venous lines were used for blood collection. CBC was performed using EDTA tubes (S-Monovette 1.2 ml, 1.6 mg EDTA/ml; SARSTEDT AG & Co., Nümbrecht, Germany). Analysis with ROTEM, ROTEM Platelet, and standard coagulation tests were performed on two different citrated tubes (S-Monovette 1.4 ml, 9NC: 0.106 mol/L, SAR-STEDT AG & Co.).

### 2.6 ROTEM thromboelastometry and FIBTEM

Rotational Thromboelastometry (ROTEM) is a viscoelastic technique that enables both graphical and numerical depiction of the coagulation process. A blood sample is placed in a cuvette into which a cylindrical pin is immersed, creating a 1 millimeter gap filled by the blood or clot. The pin rotates back and forth, moving freely when the blood is liquid. As the blood clots, the clot increasingly restricts the movement of the pin, causing the rotation to decrease as the clot becomes firmer. This change is detected optically and generates the ROTEM two-sided curve. The maximum clot firmness (MCF) and the amplitude of the curve 10 min after clotting (A10) are recorded in millimeters. In FIBTEM analysis, coagulation is initiated with tissue thromboplastin (tissue factor) and the addition of cytochalasin D blocks platelet activity. Consequently, the resulting coagulation process relies solely on fibrinogen and a lower MCF or A10 indicates abnormal clot formation, either due to reduced fibrinogen concentration or activity.

### 2.7 ROTEM platelet impedance aggregometry and TRAPTEM

The ROTEM platelet system employs impedance aggregometry, which measures changes in electrical impedance between two electrodes as platelet aggregation occurs in response to an agonist. Whole blood is placed in a cuvette with a stirring bar and electrodes set to a specific voltage. An impedance baseline is first established. Once aggregating agents are added, platelets activate and begin to aggregate. The system measures the increase in electrical impedance over time, which corresponds to the extent of platelet aggregation. The data is then analyzed to generate a curve. From the curve, the following parameters are assessed: the Area Under the Curve (AUC), which represents the total area from the start of the measurement up to 6 min; the Maximum Slope (MS) of the aggregation curve; and the Amplitude at 6 min (A6), which measures the impedance 6 min after the test begins. AUC reflects the total platelet aggregation over time. MS indicates the rate at which aggregation occurs, while A6 measures the extent of platelet aggregation at the 6-minute mark. In TRAPTEM, platelet activation is achieved using thrombin receptor activating peptide (TRAP). TRAPTEM values can be affected by drug-induced platelet dysfunction, such as with PAR-1 receptor or GP IIb/IIIa inhibitors, as well as by conditions that impair platelet aggregation, including CPB, trauma, or sepsis.

### 2.8 Laboratory analysis

Semiconductor laser flow cytometry was employed for CBC, while nephelometry was used for determining fibrinogen level and calculating prothrombin time (PT) and activated partial thromboplastin time (aPTT). The hospital laboratory performed these analyses as part of routine patient management. Thromboelastometry assessed fibrin formation and polymerization using ROTEM whole blood Haemostats System Type Delta (Axon Lab AG, Le Mont-sur-Lausanne, Switzerland). Blood samples were processed within 15 min. Fibrinogen activity was tested by placing 0.3 ml of blood in a tube containing the specific reagent, which was then transferred by pipette into the cup in contact with the rotating pin. MCF and A10 were recorded for FIBTEM analysis. Impedance aggregometry with ROTEM platelet Whole Blood Impedance aggregometry Module (Axon Lab AG, Le Mont-sur-Lausanne, Switzerland) was utilized to analyze platelet function. A citrated tube was used to draw 0.2 ml of blood, which was then placed in a cuvette with a stirring bar and electrodes. Impedance baseline was calculated for 3 min. In TRAPTEM test, aggregation was activated by the addition of TRAP. Impedance change was measured for 6 min. According to ROTEM Platelet recommendations, the following parameters were recorded: A6, MS, and AUC. All analyses were conducted by a limited number of staff members specifically trained for the study.

### 2.9 Statistical analysis

Continuous variables are expressed as medians and interquartile ranges [IQR], while categorical variables are expressed as absolute numbers and percentages N (%). Differences between pre- and post-CPB variables were analyzed using a Student *t*-test for paired samples. Groups of patients were compared using the Wilcoxon/Mann-Whitney test. Linear regression and Spearman's correlation were used to describe correlations between continuous variables. Multivariate regressions were performed to adjust for possible confounding factors. Correlations are described using beta coefficients and 95% confidence intervals (95% CI). A missing data rate <5% was considered insignificant for statistical analysis and no statistical adjustment was performed. The significance level was set at *p* = 0.05. All analyses and graphics were performed with STATA/SE 17.0.

## 3 Results

### 3.1 Study population

**Figure 1:**
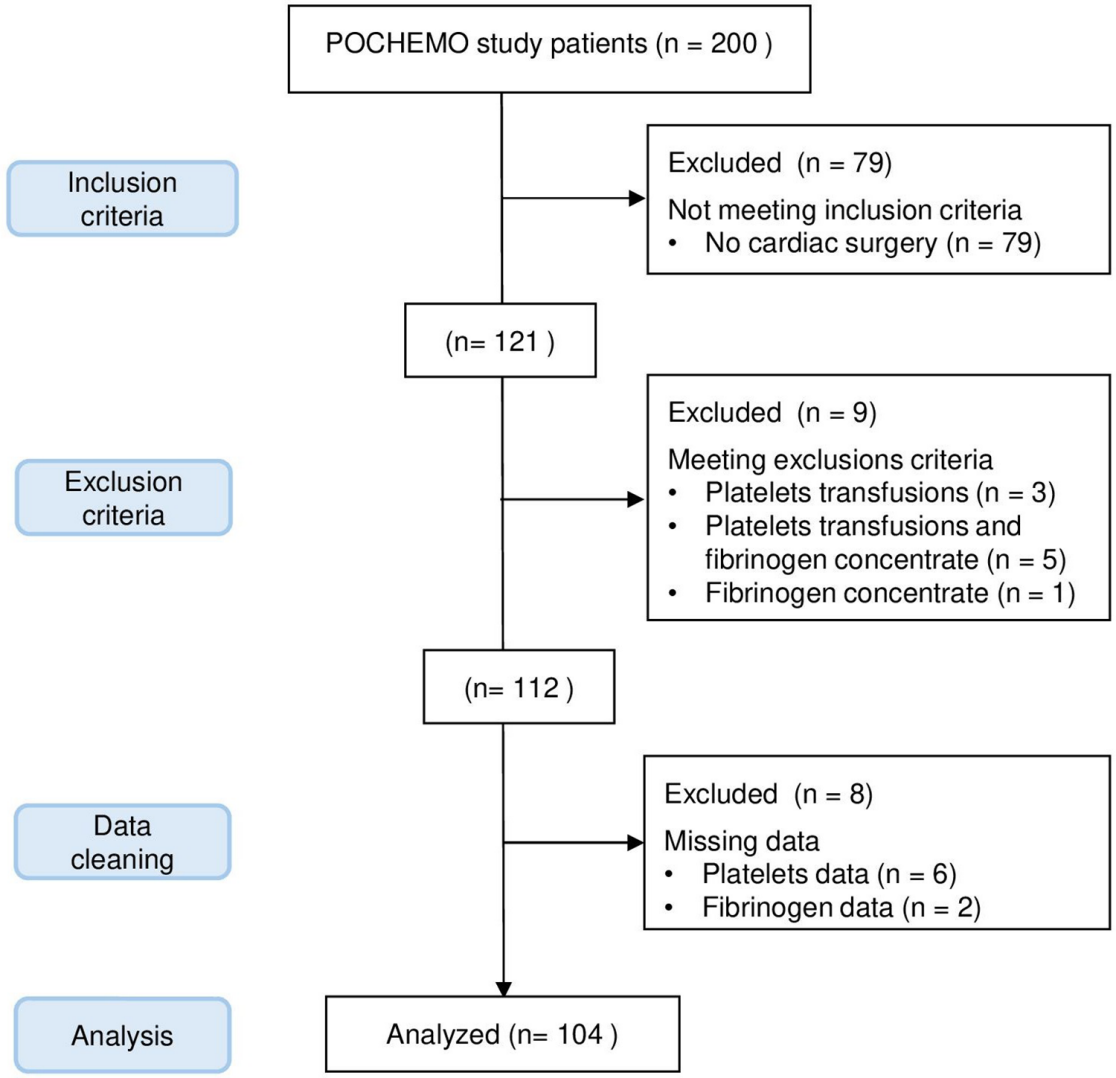
Patients inclusion flow diagram.

Out of the 200 patients in the POCHEMO cohort study, 79 patients did not undergo surgery under CPB. Among the remaining 121 patients, 17 were excluded because they received platelets or fibrinogen during surgery or because of missing data. Details are shown in the flow diagram, Figure 1. Ultimately, 104 patients were included in the final analysis. There was no variable for which more than 5% of the data were missing. The age range was 4 days old to 15 years old, with a median age of 3.4 years old [0.6–5.8] and a median weight of 12.1 kg [6.0–18.6]. Thirty-three (33%) patients had pre-operative saturations below or equal to 90%. The median hemoglobin before surgery was 130 mg/L [113–143 mg/L], and the median hematocrit was 38% [34%–42%]. Patients had a variety of heart diseases, with diagnostic categories described in Table 1.

**Table 1:** Congenital heart diseases, surgical risk categories, blood loss during and after CPB, fluid balance and FFP during surgery.

Variables		Frequency, *N*	Percentage
Congenital heart disease	VSD	27	26.0
	TOF	21	20.2
	ASD	14	13.5
	TGA	10	9.6
	AVSD	10	9.6
	PA/VSD	4	3.9
	Aortic stenosis	4	3.9
	Other	14	13.5
Congenital heart surgery	RACHS-1	4	3.9
	RACHS-2	58	55.8
	RACHS-3	39	37.5
	RACHS-4	3	2.9
Variable (ml/kg)		Median	IQR
Fluid balance at the end of CPB		6.7	(−2,3)-24.6
Blood loss during surgery		1.4	0.5–3.7
FFP during CPB		8.3	4.0–16.7
Cumulative blood loss 6 h after surgery		3.9	1.6–6.3

RACHS, risk adjustment for congenital heart surgery; VSD, ventricular septal defect, TOF, tetralogy of fallot; ASD, atrial septal defect; TGA, transposition of great arteries; AVSD atrioventricular septal defect; PA/VSD pulmonary atresia with VSD; IQR, interquartile range; CPB, cardiopulmonary bypass; FFP fresh-frozen plasma.

### 3.2 Surgical procedure and post-operative bleeding

CPB had a median duration of 112 min [84–157 min] and aortic cross-clamping of 55 min [32–85 min]. The median minimum temperature was 35.3°C [34.4–35.8°C], with 41 (39%) patients experiencing controlled hypothermia, of whom 32 in mild hypothermia (defined as 32–34.9°C), 7 in moderate hypothermia (defined as 28–31.9°C) and 2 in deep hypothermia (defined as <28°C). Eighty-six patients (83%) received fresh frozen plasma (FFP) during the procedure. Detailed data on clinical and surgical aspects and intraoperative and postoperative bleeding are provided in Table 1. Sixty-nine (66%) patients had the CPB circuit primed with crystalloids, while thirty-five received blood products. Those who received blood products priming were significantly younger (0.46 years [0.25–2.6] vs. 4.74 years [2.71–7.91], *p* < 0.001) than those who received crystalloid priming. Minimum core temperature was lower in patients primed with blood products (34.8°C [33.5–35.7] vs. 35.4°C [34.6–35.8), while there was no statistically significant difference in CPB duration (99 min [82–122] vs. 119 min [85–168], *p* = 0.16) or fluid balance at the end of CPB (9.26 ml/kg [(−21.58)-37.91] vs. 6.05 (−0.8)-23.81], *p* = 0.74). The median fluid balance at the end of CPB was positive, and blood losses were less than 1.5 ml/kg (Table 1). Only one patient experienced bleeding >7 ml/kg/h for two consecutive hours and >5 ml/kg/h on average during the 6 h after surgery. This patient was an 8-day-old neonate with a transposition of the great arteries. Four patients required a second surgical procedure within the first 24 h respectively due to right atrial compression by a clot, residual stenosis of the left pulmonary vein, low cardiac output syndrome (LCOS) requiring emergency chest opening, and severe biventricular dysfunction and LCOS needing extracorporeal membrane oxygenation (ECMO) 8 h after surgery. One patient could not be weaned of CPB and was transferred from the operating room to intensive care on ECMO. There was a significant inverse correlation between cumulative intraoperative and 6-hour postoperative blood loss and postoperative platelet count (*r* = −0.42, *p* < 0.001), postoperative percentages of baseline platelet count and fibrinogen concentration (*r* = −0.45, *p* < 0.001; *r* = −0.23, *p* = 0.02), pre-operative and postoperative TRAPTEM value (*r* = −0.24, *p* = 0.02 and *r* = −0.28, *p* = 0.004).

### 3.3 Hemostasis before and after CPB

After CPB, platelet count and fibrinogen concentration decreased in 102 (98%) and 100 (96%) patients, respectively. Thromboelastometry analyses revealed decreased fibrinogen activity in 86 (83%) patients. Impedance aggregometry revealed a drop in TRAPTEM A6 in 73 (70%) patients, TRAPTEM MS in 55 (53%) patients, and TRAPTEM AUC in 76 (73%) patients. Detailed data and median variations are presented in Table 2. There was a significant association between fibrinogen concentration and FIBTEM-MCF (*N* = 103, *r* = 0.48, *p* < 0.001) and between platelet count and TRAPTEM AUC (*N* = 102, *r* = 0.02, *p* = 0.024). Regarding the correlation between quantitative and functional analyses, the decrease in platelet count showed a significant monotonic correlation with the reduction in TRAPTEM AUC (*r* = 0.22, *p* = 0.03), although the correlation was not linear (*p* = 0.22). In contrast, the decrease in fibrinogen exhibited a strong and significant correlation with the reduction in FIBTEM MCF (*r* = 0.48, *p* < 0.001), and this correlation was also linear (*p* < 0.001).

**Table 2:** Conventional coagulation tests, ROTEM and ROTEM platelets data at baseline and after cardiopulmonary bypass (CPB).

Variables	Baseline median [IQR]	Post CPB median [IQR]	*p* value
Platelets (10^9^/L)	319.0 [237.5–393.0]	166.5 [111.5–215.0]	<0.001
Fibrinogen (g/L)	2.1 [1.8–2.4]	1.5 [1.3–1.8]	<0.001
aPTT (s)	33.0 [30.0–37.5]	38.0 [33.5–45.5]	<0.001
PR (%)	80 [70–90]	65 [60–70]	<0.001
TRAPTEM AUC (Ohm × min)	99 [73–118]	64 [48–88]	<0.001
TRAPTEM MS (Ohm/min)	9 [6–11]	6 [4–9]	0.004
TRAPTEM A6 (Ohm)	24 [18–28]	16 [12–20]	<0.001
FIBTEM MCF (mm)	10 [9–13]	8 [6–10]	<0.001

IQR, interquartile range; aPTT, activated partial thromboplastin time; PR, prothrombin ratio; TRAPTEM, impedance aggregometry test, platelets activated with thrombin receptor activating peptide (TRAP); AUC, area under the curve; MS, maximum slope; A6, amplitude at 6 min; FIBTEM, thromboelastometry test, coagulation is activated by tissue factor and platelets are blocked with cytochalasin; MCF, maximum clot firmness.

### 3.4 Conditions associated with platelets or fibrinogen impairment

**Figure 2:**
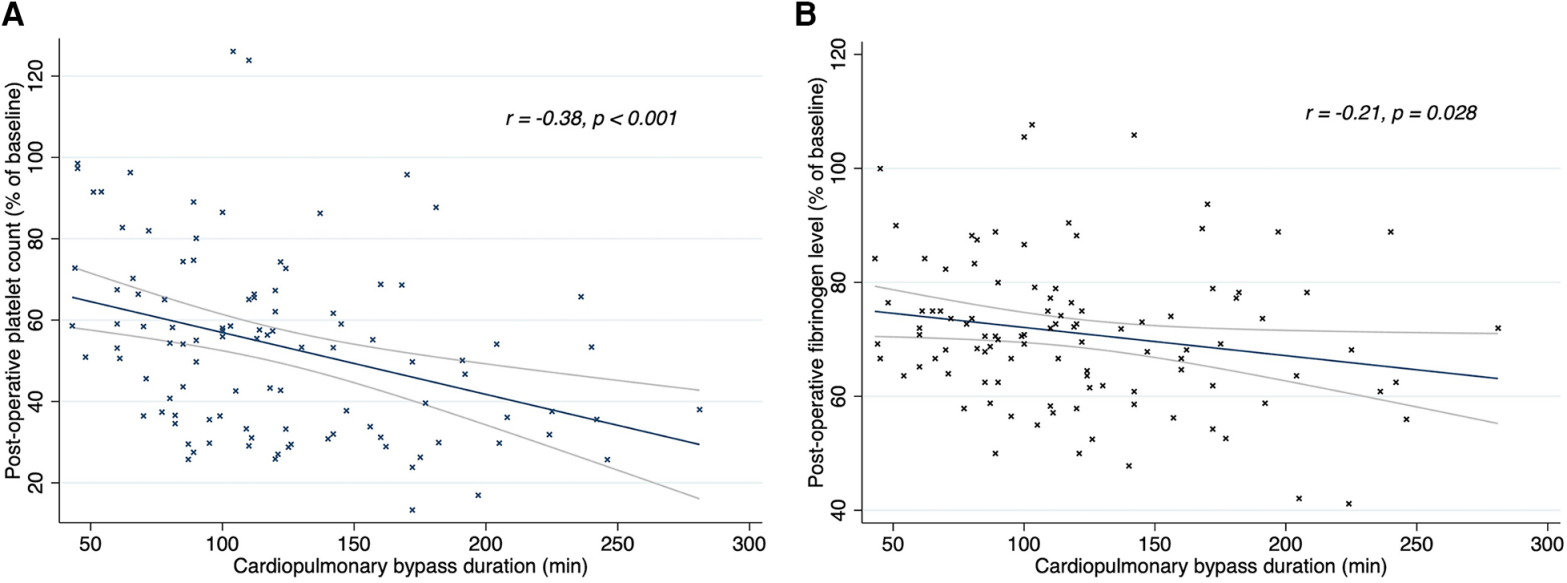
Linear regression (95% CI) and Spearman's correlations between CPB duration and platelet count **(A)** and fibrinogen level **(B)** after CPB. Post-CPB fibrinogen level and platelet count are expressed in percentage of baseline values. R, rho; CPB, cardiopulmonary bypass.

**Figure 3:**
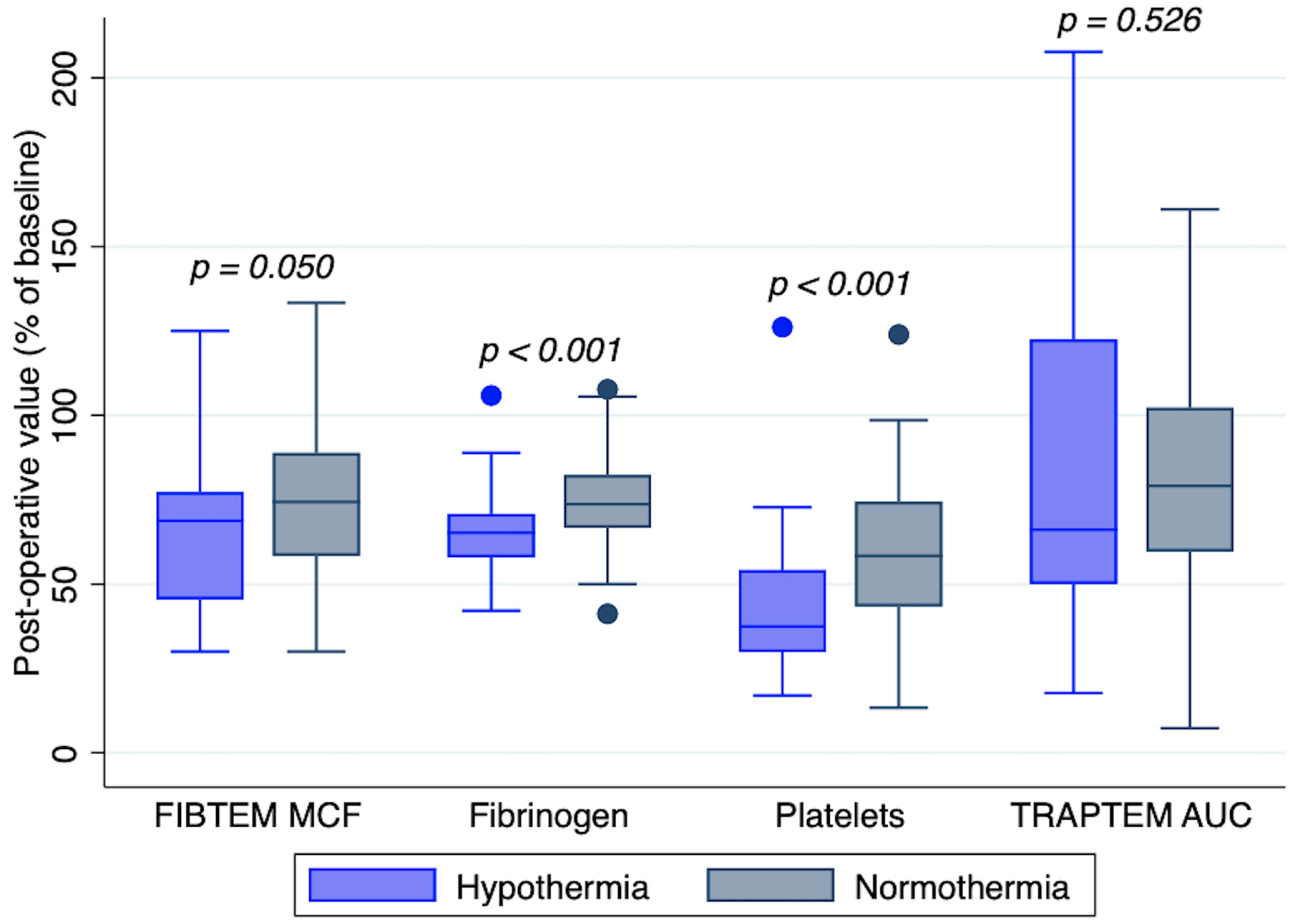
Platelet count, fibrinogen level, FIBTEM MCF and TRAPTEM AUC after cardiopulmonary bypass with normothermia or hypothermia (<35°C). Post-CPB values are expressed in percentage of baseline values. TRAPTEM, impedance aggregometry test, platelets activated with thrombin receptor activating peptide (TRAP); AUC, area under the curve; FIBTEM, thromboelastometry test, coagulation is activated by tissue factor and platelets are blocked with cytochalasin; MCF, maximum clot firmness.

**Figure 4:**
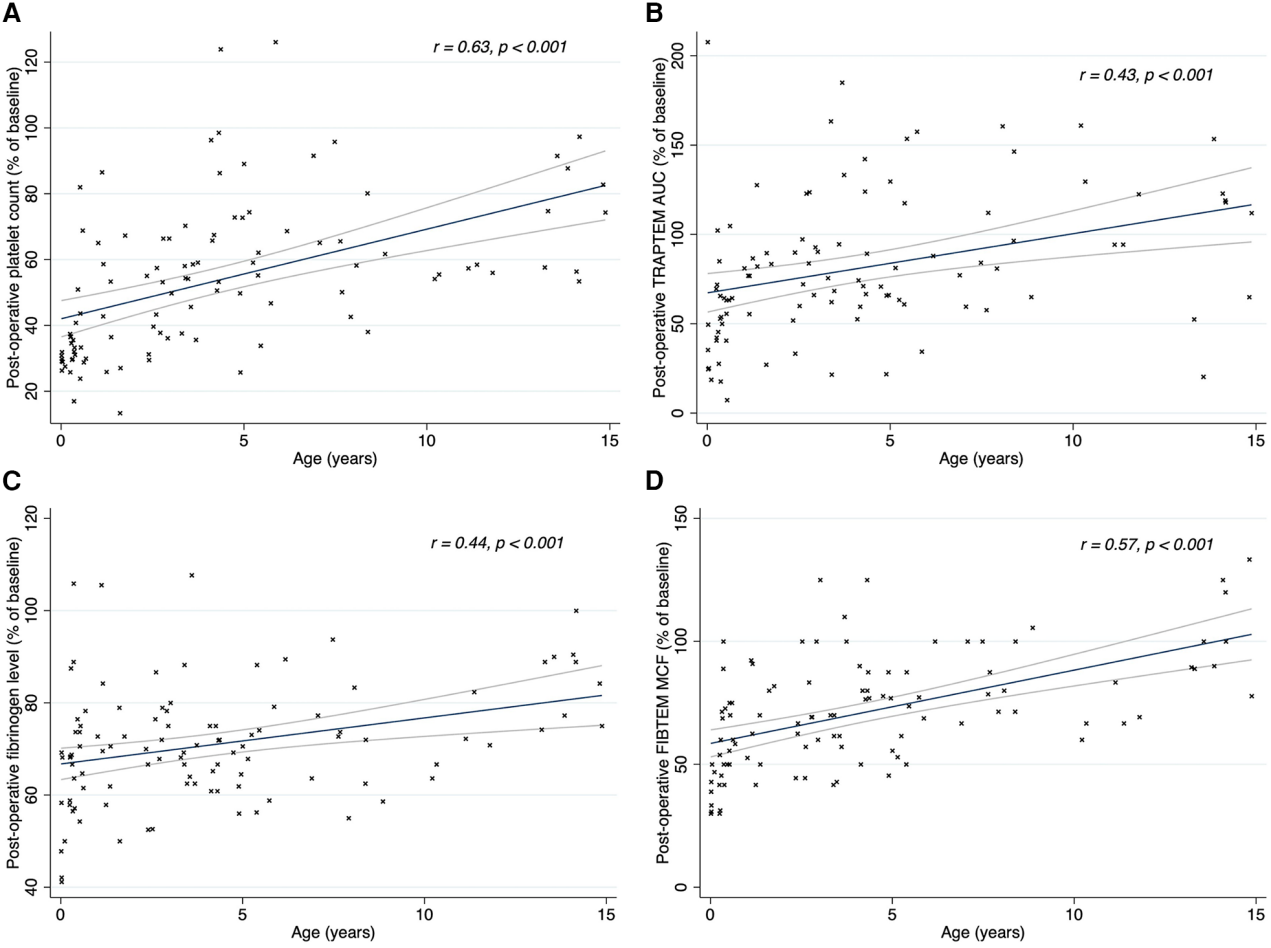
Linear regression (95% CI) and Spearman's correlations between patients’ age and platelet count **(A)**, TRAPTEM AUC **(B)** fibrinogen level **(C)** and FIBTEM MCF **(D)** after CPB. Post-CPB values are expressed in percentage of baseline values. R, rho; TRAPTEM, impedance aggregometry test, platelets activated with thrombin receptor activating peptide (TRAP); AUC, area under the curve; FIBTEM, thromboelastometry test, coagulation is activated by tissue factor and platelets are blocked with cytochalasin; MCF, maximum clot firmness.

The duration of CPB showed a negative correlation with postoperative platelet count (*r* = −0.38; *p* < 0.001) and fibrinogen concentration (*r* = −0.21; *p* = 0.028), as shown in Figure 2. There was no correlation between CPB duration and FIBTEM-MCF (*r* = 0.07; *p* = 0.461) or platelet function tests (TRAPTEM-AUC *r* = 0.11, *p* = 0.263; TRAPTEM-A6 *r* = 0.09, *p* = 0.344; TRAPTEM-MS *r* = 0.14, *p* = 0.172). Multivariate analyses confirmed that the platelet count after CPB had an inverse association with CPB duration, even after adjustment for age and hypothermia (β coefficient −0.14; 95% CI −0.21 to −0.07, *P* < 0.001). Postoperative percentages of baseline values for platelet count (58.36% [43.34–74.44] vs. 37.44% [29.81–54.17], *p* < 0.001) and fibrinogen concentration (73.68% [66.67–82.35] vs. 65.22% [57.89–70.83], *p* < 0.001) were significantly higher in patients who did not experience hypothermia (<35°C) during surgery, as shown in Figure 3. Minimum temperature showed a strong positive correlation with the fibrinogen concentration after CPB (β coefficient 1.77; 95%CI 0.47 to 3.08, *p* = 0.008) after adjustment for CPB duration and age. There was no association between the decrease in fibrinogen concentration or FIBTEM-MCF and the amount of FFP per kg received during the procedure (*r* = 0.01, *p* = 0.91 and *r* = −0.09, *p* = 0.36 respectively), even after adjusting for age, CPB duration and hypothermia. Age was inversely associated with the decrease in platelet count (*r* = 0.63, *p* < 0.001), fibrinogen concentration (*r* = 0.32, *p* < 0.001), and functional analyses (TRAPTEM-AUC *r* = 0.44, *p* < 0.001; FIBTEM-MCF *r* = 0.57, *p* < 0.001), as shown in Figure 4. When adjusted for CPB duration and degree of hypothermia, age showed a significant positive association with the postoperative percentage variation of platelet count (β coefficient 2.55, 95% CI 1.69–3.42, *p* < 0.001), fibrinogen concentration (β coefficient 0.84, 95% CI 0.27–1.41, *p* = 0.004), TRAPTEM-AUC (β coefficient 13.75, 95% CI 4.50–22.99, *p* = 0.004) and, FIBTEM-MCF (β coefficient 2.77, 95% CI 1.81–3.74, *p* < 0.001).

There was no association between the decrease in fibrinogen concentration or FIBTEM-MCF and the amount of FFP per kg received during the procedure, once adjusted for age, CPB duration, and hypothermia. There was no association between the fluid balance after CPB and conventional (platelet count *r* = −0.1, *p* = 0.297; fibrinogen level *r* = 0.01, *p* = 0.957) or functional coagulation tests (TRAPTEM-AUC *r* = −0.17, *p* = 0.08; FIBTEM *r* = −0.06, *p* = 0.496). Postoperative percentages of baseline values for platelet count (58.08% [42.63–72.85] vs. 36.49% [29.82–50.95], *p* < 0.001), TRAPTEM AUC (80.95% [64.38–119.05] vs. 63.21% [42.27–86.67], *p* = 0.009), fibrinogen concentration (72.73% [63.64–79.17] vs. 68.18% [58.33–72.00], *p* = 0.018), and FIBTEM MCF (72.73% [63.64–79.17] vs. 68.18% [58.33–72.00], *p* = 0.018) were significantly higher in patients who received crystalloids for priming compared to those who received blood products.

## 4 Discussion

This study, based on prospective data from a large pediatric cohort, excludes patients who received platelet transfusions or fibrinogen concentrate during CPB. Our data confirm that fibrinogen concentration and platelet count are significantly reduced after CPB, as are their specific activities measured by thromboelastometry and impedance aggregometry. This decrease correlates with CPB duration and patients’ age. In contrast, we did not observe the platelet hyper-reactivity described by other authors in post-CPB.

A decrease in platelet count following CPB has been extensively described in various pediatric and neonatal cohorts, and our study confirms this phenomenon (5, 8, 11, 18, 19). There are limited and contradictory data regarding platelet activity after cardiac surgery. Similar to other studies in the pediatric population, we observed a significant decrease in platelet activity measured by impedance aggregometry (8, 10, 19). Andreasen et al. reported a similar reduction but with a paradoxical increase in platelet count and function after shorter CPB durations (less than 100 min) (11). In newborns, Zwifelhofer et al. described a decrease in platelet reactivity to stimulation by U46619, collagen-related peptide, adenosine diphosphate, and TRAP during CPB, which rapidly corrected after CPB. The TRAP-induced stimulation was already normalized post-CPB, and only ADP response remained impaired upon arrival in the intensive care unit (5). It is worth mentioning that all these patients had received platelet transfusions before the end of CPB. Ignjatovic et al. described postoperative thrombocytopenia associated with platelet hyperactivity in a pediatric population based on beta-thromboglobulin overexpression. In this case, approximately 50% of patients had received platelets during the procedure (18). Our study observed a persistently impaired platelet response to TRAP at the end of the procedure. In the aforementioned studies, platelet transfusion during the procedure could have led to faster platelet activity recovery and even transient hyperactivity. This hypothesis is supported by Barker and colleagues’ study, which showed a significant drop in platelet activity, measured by thromboelastography, only in the cohort of patients who did not receive an intraoperative platelet transfusion (10).

The drop in platelet count and function post-CPB has multiple explanations. On one hand, there is undoubtedly some hemodilution at the beginning of the procedure, during the priming phase, and throughout the entire CPB to maintain fluid balance. On the other hand, increased platelet consumption is likely due to several factors: activation of the hemostatic system upon contact with synthetic surfaces, mechanical damage to platelets from shear stress within the CPB circuit, systemic inflammation induced by CPB, and hemostasis at the surgical site (20). Moreover, our study indicates that the decrease in TRAPTEM AUC does not exhibit a linear correlation with the reduction in platelet count, suggesting that the same processes responsible for platelet consumption during CPB may also impair the function of the remaining circulating platelets.

There is less data regarding the CPB-induced changes in fibrinogen concentration and activity. Hayashi et al. described a postoperative increase in fibrinogen level; Andreasen et al. did not show significant changes, while other authors reported a decrease (7, 11, 21, 22). Our study found a significant reduction in fibrinogen concentration and FIBTEM MCF. The decrease in fibrinogen concentration is likely due to increased consumption, for similar reasons to those mentioned above for platelet consumption, such as hyperactivation of the hemostatic system and surgical wounds healing. In addition, contact with the CPB circuit induces increased fibrinolysis, which further contributes to fibrinogen consumption (20). The variability in these results could be attributed to different blood product administration practices during CPB and different sampling timing. Hayashi et al. collected samples 24 h after CPB, which may have allowed complete correction of hypofibrinogenemia, along with a fibrinogen increase due to postoperative inflammation. These studies have not described the correlation between fibrinogenemia and the dose of FFP or cryoprecipitate received during the procedure. We found no association between the amount of FFP administered during the procedure and the fall in fibrinogen concentration. However, FFP contains a much lower concentration of fibrinogen than cryoprecipitate (2.5 g/L vs. 15 g/L), which is used in other centers during CPB (23).

Regarding the variables associated with post-CPB coagulopathy, lower core temperature has been associated with a more significant decrease in platelet count and activity and increased postoperative bleeding (5, 11, 24). *in vitro*, hypothermia reduces platelets aggregation and adhesion, as well as fibrinogen synthesis and fibrin network (25, 26). We observed a correlation between hypothermia and decreased fibrinogen concentration and platelet count, but not in their activity measured by FIBTEM MCF and TRAPTEM AUC. It should be highlighted that in our cohort, most of the patients were normothermic, and only 9% were in moderate to severe hypothermia. The duration of CPB has also been widely linked to a more pronounced decrease in platelet count and increased postoperative bleeding (7, 8, 11, 21, 27–29). Considering the aforementioned pathophysiological processes, it is not difficult to imagine how prolonged CPB could lead to increased consumption of platelets and fibrinogen. We found a strong correlation between CPB duration and the reduction of fibrinogen level and platelet count but not with the activities measured by ROTEM. Finally, as already pointed out by other authors, our analysis confirms a negative correlation between age and the decline in platelet count, fibrinogen concentration, and their activities (1, 18, 19, 30). In our study, despite priming with blood products, younger children exhibited more pronounced coagulopathy, possibly due to lower core temperatures during CPB or a proportionally higher exposure of circulating blood to the CPB circuit. Mainly, precise surgical hemostasis and careful monitoring of functional coagulation tests with rapid correction of abnormalities are critical in infants.

In our cohort, only one patient met the most recent criteria for excessive bleeding, according to Bercovitz et al. (3). Even when considering different cutoffs proposed by other authors, such as 10% of circulating volume during the first 6 h or >5 ml/kg/h during the first hour, we identified only five patients with significant bleeding (3, 21, 29, 31). In the entire POCHEMO cohort, there was another patient who met Bercovitz's criteria for excessive bleeding, but he was excluded from secondary analyses because he received platelet transfusions during CPB. We have no definitive explanation for the minimal bleeding observed in our cohort. Still, it may be related to the CPB strategy of maintaining normothermia or mild hypothermia as often as possible. Additionally, careful surgical hemostasis and broad use of FFP while weaning CPB may have played a significant role.There appears to be a link between the disturbance in hemostatic parameters and hemorrhagic diathesis. In adults, this relationship is well defined; post-CPB bleeding correlates with thrombocytopenia, hypofibrinogenemia, and the percentage decrease in fibrinogen levels after CPB (32). Furthermore, in adults, thromboelastography or thromboelastometry-based coagulation management in cardiac surgery reduces the need for transfusion and the re-exploration rate (33). In children, this relationship is less obvious. Despite the minimal bleeding we observed, our data suggest a correlation between intra- and post-operative blood loss and the drop in platelet count and fibrinogen concentration. Several authors have shown a correlation between thrombocytopenia, a percentage decrease in platelet count or abnormal response to TRAP stimulation, and postoperative bleeding (7, 8, 19, 34). However, according to Zwifelhofer et al., thrombocytopenia and platelet dysfunction do not correlate with postoperative bleeding, and platelet transfusion normalizes quantitative and functional tests but does not correct the bleeding diathesis (5).

An association between fibrinogen concentration or its decrease and postoperative bleeding appears well established (21, 31, 35). A cutoff of 1.5 g/L has been proposed for fibrinogenemia, with lower values associated with excessive postoperative bleeding (30, 35). In our cohort, 48 (46%) children had postoperative fibrinogen levels lower than 1.5 g/L. Still, the FIBTEM MCF median value was within the normal pediatric range, according to Oswald et al. (36). We had only one significant bleeding event, supporting FIBTEM's superiority in assessing postoperative bleeding risk, as others already pointed out by others (21, 27, 28, 37). In contrast, Pekelharing et al. did not demonstrate an additional benefit of thromboelastography over standard coagulation tests in the postoperative period (31). However, further support for a clinical correlation between thromboelastometry and postoperative bleeding is provided by studies on ROTEM-guided hemostasis correction algorithms, which seem to reduce the need for transfusion and postoperative blood loss also in pediatric patients (13, 14, 37).

To our knowledge, this is the largest study focused on CPB's quantitative and functional effects on platelets and fibrinogen in children. Furthermore, we excluded all patients who had received intraoperative platelet transfusions or fibrinogen concentrate to observe with greater precision the impact of CPB on these components of hemostasis. Another strength of our study is that it is based on prospectively collected, robust data from a pediatric cohort of various ages and pathologies, and it has very few failing tests. Additionally, we performed quantitative and functional analyses using impedance aggregometry (ROTEM Platelet) and thromboelastometry (FIBTEM by ROTEM) for each patient. Our study also has several limitations; we only have measurements taken immediately before and after CPB and not during or several hours after surgery, which would have allowed for a more precise analysis of variable changes over time. Clinical follow-up data are limited to the first 6 h post-surgery, which limits prognostic assessment. Furthermore, as previously discussed, we could not correlate our findings with postoperative bleeding.

Post-CPB coagulopathy is a complex phenomenon that mainly affects young children and patients who undergo prolonged CPB and hypothermia. This condition is not only caused by hemodilution but also involves changes in the function of components in the coagulation cascade, which can be identified through thromboelastometry and impedance aggregometry.

## Data Availability

The data analyzed in this study is subject to the following licenses/restrictions: The study is a secondary analysis based on data collected by some of the authors during the POCHEMO study. Data were anonymized and stored in a database. Raw data will be made available on request, specifying the purpose of the demand. Requests to access these datasets should be directed to margherita.plebani@chuv.ch.
